# Precise Topographic Model Assisted Slope Displacement Retrieval from Small Baseline Subsets Results: Case Study over a High and Steep Mining Slope

**DOI:** 10.3390/s20226674

**Published:** 2020-11-21

**Authors:** Lianhuan Wei, Qiuyue Feng, Feiyue Liu, Yachun Mao, Shanjun Liu, Tianhong Yang, Cristiano Tolomei, Christian Bignami, Lixin Wu

**Affiliations:** 1 Institute for Geo-Informatics and Digital Mine Research, School of Resources and Civil Engineering, Northeastern University, Shenyang 110819, China; 1800987@stu.neu.edu.cn (Q.F.); maoyachun@mail.neu.edu.cn (Y.M.); liushanjun@mail.neu.edu.cn (S.L.); 2 Center for Rock Instability and Seismicity Research, School of Resources and Civil Engineering, Northeastern University, Shenyang 110819, China; 1510444@stu.neu.edu.cn (F.L.); yangtianhong@mail.neu.edu.cn (T.Y.); 3 Istituto Nazionale di Geofisica e Vulcanologia, 00143 Rome, Italy; cristiano.tolomei@ingv.it (C.T.); christian.bignami@ingv.it (C.B.); 4 School of Geosciences and Info-Physics, Central South University, Changsha 410083, China; wulx66@csu.edu.cn

**Keywords:** open-pit mine, high and steep slope, landslide monitoring, small baseline subsets analysis, digital elevation model

## Abstract

Due to the intrinsic side-looking geometry of synthetic aperture radar (SAR), time series interferometric SAR is only able to monitor displacements in line-of-sight (LOS) direction, which limits the accuracy of displacement measurement in landslide monitoring. This is because the LOS displacement is only a three dimensional projection of real displacement of a certain ground object. Targeting at this problem, a precise digital elevation model (DEM) assisted slope displacement retrieval method is proposed and applied to a case study over the high and steep slope of the Dagushan open pit mine. In the case study, the precise DEM generated by laser scanning is first used to minimize topographic residuals in small baseline subsets analysis. Then, the LOS displacements are converted to slope direction with assistance of the precise DEM. By comparing with ground measurements, relative root mean square errors (RMSE) of the estimated slope displacements reach approximately 12–13% for the ascending orbit, and 5.4–9.2% for the descending orbit in our study area. In order to validate the experimental results, comparison with microseism monitoring results is also conducted. Moreover, both results have found that the largest slope displacements occur on the slope part, with elevations varying from −138 m to −210 m, which corresponds to the landslide area. Moreover, there is a certain correlation with precipitation, as revealed by the displacement time series. The outcome of this article shows that rock mass structure, lithology, and precipitation are main factors affecting the stability of high and steep mining slopes.

## 1. Introduction

Open-pit mining is one of the most widely used methods for ore excavation in China. With the continuous increase of mining depth, most open-pit mines initiate deep concave mining modes, resulting in increased slope height and slope angles (and, unfortunately, decreased slope stability). In deep open pit mines, stability of the high and steep slopes plays an important role for the safety of mine production. Instead of occurring immediately after ore excavation, most landslides of high and steep slopes occur after a period of time. Under long-term effects of excavation, rock mass loading, and high permeability, the mining slopes usually suffer from gradual displacement, structural failures, e.g., creep, cumulative damage, dynamic load fatigue, and elastic mutation, etc. Therefore, the measurement of surface displacement is vital for understanding landslide evolution mechanisms and early warning of catastrophic failures [1].

However, it is difficult to measure displacement of landslides by ground survey for high and steep slopes, due to the imperceptibility, unpredictability, and inaccessibility of many landslides. Moreover, ground survey measurements can only provide accurate displacement at a limited number of points, instead of spatial distribution patterns of displacements over the whole area. As an important complement to traditional ground survey methods, synthetic aperture radar interferometry (InSAR) has become a popular tool for landslide monitoring, especially with the rapid development of time series InSAR (TS-InSAR) algorithms, such as permanent scatterer interferometry (PSI), small baseline subsets (SBAS) analysis, and some hybrid methods [2,3,4,5,6,7,8,9]. The first application of spaceborne differential InSAR to landslide investigation was conducted in 1996 [2]. However, several successful landslide monitoring examples only started to draw attention of the landslide community after the development of PS-InSAR and similar TS-InSAR methods [10,11,12]. With the continuous progress in SAR data processing techniques and new generation radar satellites (e.g., TerraSAR-X/TanDEM-X, COSMO-SkyMed, Radarsat-2, ALOS PALSAR-1/2, Sentinel-1A/B, etc.), many new landslide monitoring results with improved spatiotemporal resolution and wide coverage have been reported by the InSAR community [13,14,15,16,17,18,19,20,21,22,23]. There are many technical and practical issues that have to be properly considered when applying TS-InSAR approaches in landslide studies, e.g., selection of SAR data sources, visibility of the landslide in SAR images, selection of enough point targets, proper handling of phase errors, geocoding accuracy, displacement model, geometric distortions, and assessment of result quality, etc. [19]. However, even if most of the above-mentioned issues have been tackled, the claimed centimeter to millimeter level accuracy of TS-InSAR results is still hardly achieved for landslide applications. Due to the intrinsic side-looking geometry of SAR sensors, TS-InSAR is only able to measure displacements in the satellite’s line-of-sight (LOS), which can be considered as a three dimensional projection of real displacement [24,25]. In fact, displacement of landslides may occur in various directions due to topographic variety. This makes interpretation of TS-InSAR measurements challenging, and limits the accuracy of displacement measurements.

Targeting at this problem, different methods were developed to resolve three-dimensional surface displacements, such as fusion of ascending and descending LOS measurements, combining LOS measurements with azimuth measurements derived by offset-tracking (OT) or multi-aperture interferometry (MAI) [25,26,27,28,29]. Unfortunately, the above-mentioned approaches all have their drawbacks. Fusion of ascending and descending LOS measurements is only applicable to areas where multiple datasets are available, and high precision in the north-south direction is only possible for high-latitude regions. OT and MAI are insensitive to slow displacements, and suffer from limited accuracy in the north–south direction as well. In deep open-pit mines, landslides frequently occur along the high and steep slopes towards bottom of the stope. Due to the various slope angles and slope aspects, converting LOS measurements to slope directions is a key problem affecting the precision of displacement monitoring using TS-InSAR.

In this article, a novel approach that resolves slope displacements from LOS measurements with assistance of a precise digital elevation model (DEM) is proposed. The precise DEM is generated by laser scanning, and LOS measurements are retrieved by SBAS processing on sentinel-1 images. The proposed method is applied to a case study in the Dagushan Deep open pit, where a landslide occurred shortly after our monitoring period. The slope displacements are resolved for both ascending and descending orbits, and compared with total station measurements and microseism monitoring results, respectively. As a result, estimated slope displacements on the ground measurement points reach relative root mean square errors (RMSE) of approximately 12–13% for the ascending orbit, and 5.4–9.2% for the descending orbit in our study area. Moreover, the results from the time series InSAR technique and microseism monitoring have found that the largest slope displacements occur on the slope part with elevations vary from −138 m to −210 m, which corresponds to the landslide area.

## 2. Methodology

### 2.1. Small Baseline Subsets Analysis

Current TS-InSAR approaches are generally categorized into two groups. The first group uses interferograms generated with reference to a common master, e.g., the traditional PSI approach [3,4]. The second group uses only high-quality interferograms generated from all possible image pairs, e.g., the SBAS approach [5,6]. While PSI focuses on displacement estimation for persistent scatterers (pixels dominated by a single scatterer), the SBAS method focuses on displacement estimation for distributed scatterers (pixels without any dominant scatterers). In PSI, the common master strategy makes low-quality interferograms with long spatiotemporal baselines participate in deformation estimation, resulting in low target density in areas with few artificial features [3,4]. In SBAS, all of the high-quality interferometric pairs with short spatiotemporal baselines are used, ensuring high temporal sampling and high point density. Benefiting from the short spatiotemporal baselines, the influence of temporal decorrelation, spatial decorrelation, and DEM error on deformation estimation are all reduced [5,6]. Therefore, the SBAS method is used for time series analysis over the study area.

In SBAS, a master image is first selected according to the spatiotemporal baseline distribution, and all other images are registered with reference to the master. Secondly, high-quality differential interferograms are generated with given thresholds on spatial and temporal baselines following a multi-master strategy. Thirdly, after phase unwrapping of the time series differential interferograms with Delaunay minimum cost flow algorithm, unwrapped interferograms are combined with Singular Value Decomposition (SVD) to generate interferometric time-series. The unwrapped phase of each pixel could be divided into components from ground displacement, topographic residuals, atmospheric disturbance, orbital error, and noise. By applying iterative filtering in spatiotemporal domains, these different components could be successfully separated, and the deformation in LOS is finally retrieved [5,6].

### 2.2. Conversion from LOS Displacements to Slope Direction

**Figure 1:**
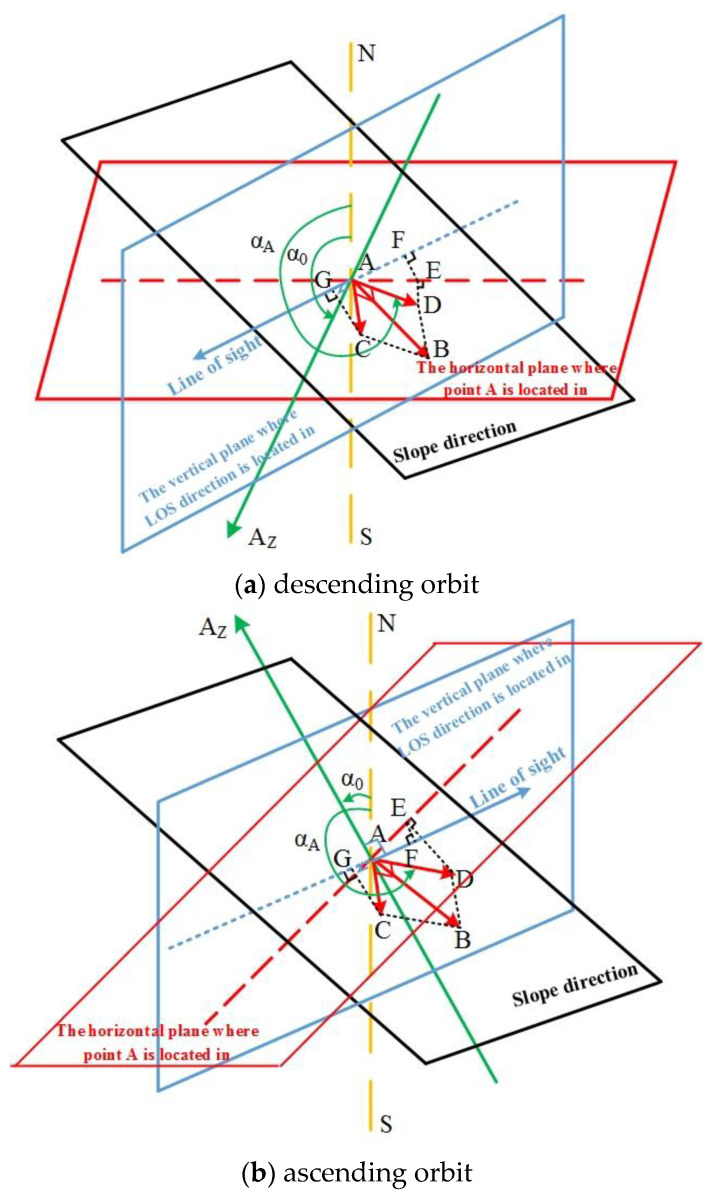
Geometric relationship between slope displacement and displacement in line-of-sight (LOS) in descending (**a**) and ascending (**b**) orbits.

In order to retrieve precise displacements of ground targets, displacements in LOS need to be converted to slope direction. The geometric relationship between deformation in LOS and slope direction is given in Figure 1, where the geometries in descending and ascending orbits are given separately. Taking point A in Figure 1a as an example, its displacement along slope direction can be divided into vertical and horizontal components, as given by the following equation:

(1) $$d_{A\_v}{=d}_{A}\cdot \sin\left(\beta \right)\;d_{A\_h}{=d}_{A}\cdot \cos\left(\beta \right)$$

where $d_{A}$ is the displacement along slope direction, β is the slope angle, $d_{A\_v}$ and $d_{A\_h}$ are the vertical and horizontal components of $d_{A}$ respectively. The LOS deformation of point A ($d_{A\_los}$) is composed of projection of $d_{A\_v}$ in LOS and projection of $d_{A\_h}$ in LOS. The projection of $d_{A\_v}$ in LOS direction can be calculated directly according to the incidence angle θ. In order to resolve the projection of $d_{A\_h}$ in LOS direction, $d_{A\_h}$ should be first projected to the vertical plane where LOS is in [30]. Since the LOS directions in descending and ascending orbits are different, the projections would be discussed separately.

In descending geometry, the projection of $d_{A\_h}$ on the vertical plane where LOS is in can be expressed as:

(2) $$d_{A\_h-los}=d_{A\_h}\cdot \sin\left(\alpha_{A}-\alpha_{0}\right)$$

where $d_{A\_h-los}$ is the projection of $d_{A\_h}$ on the vertical plane where LOS is in, $\alpha_{A}$ is the counter-clockwise angle between $d_{A\_h}$ and north direction, $\alpha_{0}$ is the heading angle of SAR sensor. Concerning the incidence angle of θ, displacement of point A in LOS ($d_{A\_los}$) is the vector summarization of the projection of $d_{A\_v}$ in LOS and the projection of $d_{A\_h-los}$ in LOS, which can be expressed as:

(3) $$d_{A\_los}=d_{A\_h-los}\cdot \sin\left(\theta \right)-d_{A\_v}\cdot \cos\left(\theta \right)$$

By substituting Equations (1) and (2) into (3), we can get:

(4) $$d_{A\_los}{=d}_{A}\cdot \cos\left(\beta \right)\cdot \sin\left(\alpha_{A}-\alpha_{0}\right)\cdot \sin\left(\theta \right)-d_{A}\cdot \sin\left(\beta \right)\cdot \cos\left(\theta \right)$$

Therefore, in descending geometry, LOS displacement of point A can be converted into slope direction with the following equation:

(5) $$d_{A}=\frac{d_{A\_los}}{\cos\left(\beta \right)\cdot \sin\left(\alpha_{A}-\alpha_{0}\right)\cdot \sin\left(\theta \right)-\sin\left(\beta \right)\cdot \cos\left(\theta \right)}$$

Similarly, in ascending geometry (Figure 1b), projection of $d_{A\_h}$ in the vertical plane where LOS is in can be expressed as:

(6) $$d_{A\_h-los}=d_{A\_h}\cdot \sin\left(\alpha_{A}-\alpha_{0}-\pi \right)=-d_{A\_h}\cdot \sin\left(\alpha_{A}-\alpha_{0}\right)$$

where $d_{A\_h-los}$ is projection of $d_{A\_h}$ in the vertical plane where LOS is in. Displacement of point A in LOS ($d_{A\_los}$) is the vector summarization between projection of $d_{A\_v}$ in LOS and projection of $d_{A\_h-los}$ in LOS, which can be expressed as:

(7) $$d_{A\_los}=-d_{A\_h-los}\cdot \sin\left(\theta \right)-d_{A\_v}\cdot \cos\left(\theta \right)$$

By substituting Equations (1) and (6) into Equation (7), the relationship between $d_{A}$ and $d_{A\_los}$ in ascending geometry can be described, which is identical to Equation (4). Therefore, for ascending data, the conversion of displacement from LOS to slope direction is the same as Equation (5) [30]. In this study, the heading angles $\alpha_{0}$ (assuming clockwise with reference to the North is positive) and incidence angles θ of the two datasets are shown in Table 1. The slope angles are derived from the precise DEM, which is approximately 47° for the landslide area.

## 3. Study Area and Data Collection

### 3.1. Geological and Hydrological Setting of the Study Area

**Figure 2:**
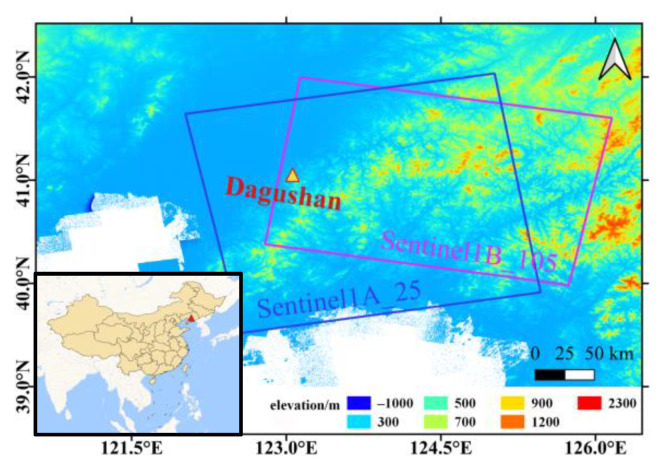
Location of Dagushan Iron Mine, with the TanDEM-X 90 m DEM as background.

Dagushan iron mine, located in northeast China (Figure 2), is a typical deep open-pit mine and one of the deepest open-pit iron mines in Asia. The deep open pit mining started from 1970, forming an elliptical pit with length of 1620 m from east to west, width of 1560 m from north to south, and a vertical depth of 450 m until 2019 [31]. The mining area has experienced multiple tectonic movements and magmatic activities, resulting in a complicated structure dominated by composite syncline with northwest trending axial and west trend. According to investigation, there are more than 50 faults and joints crisscrossing in the mining stope, and more than 30 dikes intrude along the pre-existing faults [32].

**Figure 3:**
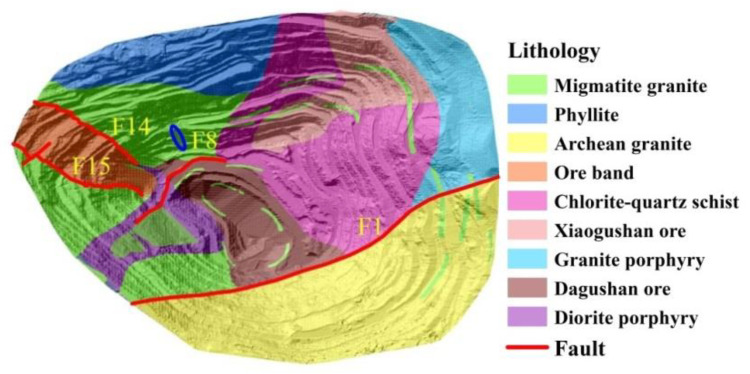
Distribution of faults and lithology in the Dagushan mining pit.

The distribution of faults and lithology in Dagushan mining pit is shown in Figure 3, where solid red lines indicate faults in the mining area. F15 is the only normal strike fault in the mining area, with occurrence of 45~55°∠70~75°. F14 is a large oblique fault with occurrence of 190~200°∠50~60°. Due to the double cutting of F15 and F14, western part of the ore body is wedge-shaped. F8 is located in the middle of the mining area, with a northeast strike (30~45°) and nearly vertical dip [33]. F1 is the largest fault in the mining area, which is basically the boundary between Archean granite and migmatite granite, with a nearly EW strike direction [34]. As shown by Figure 3, the lithology exposed in the mining stope includes migmatite granite, phyllite, Archean granite, chlorite quartz schist, granite porphyry, diorite porphyry, and iron ore band as well [35]. The annual precipitation in the mining area is 720 mm on average, most of which happens during summer and autumn. The ground water in the Dagushan area consists of surface groundwater, pore water in the loose rocks, and fissure water in the bedrocks [36]. Weak, water-rich, and fractured aquifer of quartzite is distributed in the northwest of the stope, especially in the chlorite quartz schist area.

**Figure 4:**
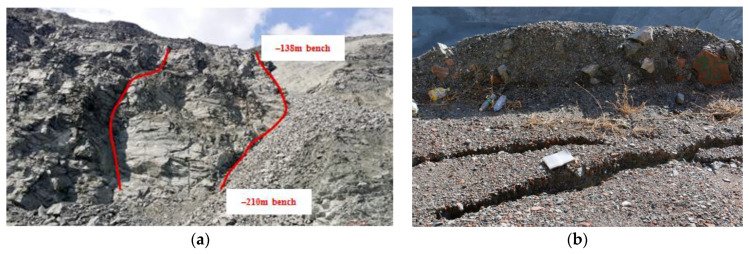
Photograph of the landslide on 27 May 2018: (**a**) left and right borders; (**b**) crack on the top border at −138 m bench.

For open pit mines, low stripping-ratio indicates high production rate, and the slope angles are therefore critically designed by numerical simulations, which aim at finding a trade-off between stripping ratio and slope stability. Moreover, different from natural slopes, mining slopes have a step-like structure with multiple wide benches and narrow steps. The wide benches are used as safety platforms, which prevent large-scale landslides, and narrow steps are designed between two benches. Therefore, small-scale landslides usually happen between two benches in open pit mines. As shown in Figure 3, the step-like structure is described in detail by the Light Detection and Ranging (LiDAR) DEM, of which the spatial resolution is 1.6 m. On 27 May 2018, a landslide with height of approximately 72 m occurred on the northwest slope, as highlighted by the blue elliptical area in Figure 3. The landslide body is located between the −138 m bench and the −210 m bench, with width of approximately 20 m [31]. Photograph of this landslide is given in Figure 4.

### 3.2. Data Collection

In this study, two stacks of Sentinel-1 images are collected to investigate the surface displacements of the Dagushan iron mine. Spatial coverage of the datasets is depicted in Figure 2, and detailed parameters are summarized in Table 1. For the study area, an ascending stack of 28 Sentinel-1A images acquired during 5 June 2017–19 May 2018 are collected, whereas a descending stack of 29 Sentinel-1B images acquired during 4 June 2017–18 May 2018 are collected.

**Figure 5:**
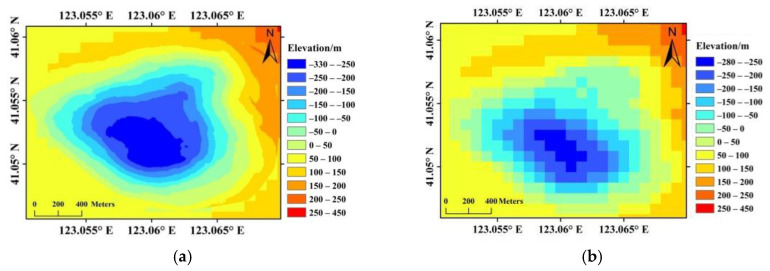
Comparison between the LiDAR DEM (**a**) and the TanDEM-X 90 m DEM (**b**).

In SBAS processing, DEM is used to simulate and remove the topographic phase component from interferograms. A high resolution DEM is able to describe details of the topography, which is necessary for precise simulation of the topographic phase component in areas with big topographic relief, and thus leads to minimum topographic residue in the estimated displacements. Therefore, a high-resolution DEM is necessary for precise LOS displacement retrieval. Besides, in areas with high topographic inequality, displacement of landslides may occur in various directions due to the diversity of slope aspects and slope angles. It is difficult to build the 3D geometry between LOS direction and slope direction with low resolution DEM. This is one of the key problems, which limits the accuracy of time series InSAR results in landslide monitoring applications. For the conversion from LOS displacement to slope direction, high-resolution DEM is able to give precise slope aspects and slope angles, and precise slope displacements are therefore retrieved. Since high resolution LiDAR DEM is only available for the mining pit instead of the whole study area, the LiDAR DEM is mosaicked with TanDEM-X 90 m DEM. The spatial resolution of LiDAR DEM is approximately 1.6 m, whereas the spatial resolution of TanDEM-X DEM is 90 m, as shown in Figure 5 [36,37]. Due to the difference in spatial resolution, the TanDEM-X DEM is first over-sampled to have equal resolution with LiDAR DEM, and then the elevation values in the open pit is replaced by elevation in the precise LiDAR DEM.

**Figure 6:**
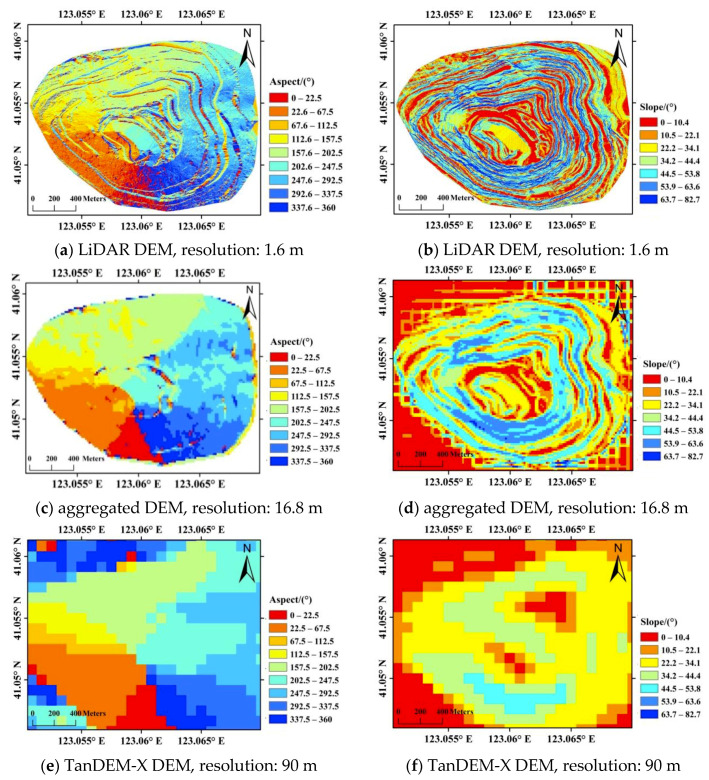
Slope aspects and angles derived from the LiDAR DEM (**a**,**b**), aggregated DEM (**c**,**d**), and TanDEM-X DEM (**e**,**f**), respectively.

However, this does not necessarily mean that higher resolution of DEM results in higher precision of slope displacements. On the other hand, as given by Figure 6a,b, the high resolution DEM (with resolution of 1.6 m) is a bit noisy for displacement conversion. Therefore, the original LiDAR DEM is aggregated to a DEM with spatial resolution of approximately 16.8 m, which is similar to the spatial resolution of sentinel-1 images. The slope aspects and angles are given in Figure 6c,d. In the aggregated DEM, the slope angles along northwest slope varies from 28° to 55°, and approximately 47° for the landslide area, which is identical with the designed mining slope angle given by reference [31]. For comparison, slope aspects and slope angles of the TanDEM-X DEM are also given in Figure 6e,f, from which we can see that the low resolution of 90 m is clearly not enough for topographic phase removal, and for displacement conversion from LOS to slope direction as well. In our experience, DEM with similar resolution to the SAR images is preferred.

## 4. Experimental Results Analysis

### 4.1. Displacement along Slope Direction

**Figure 7:**
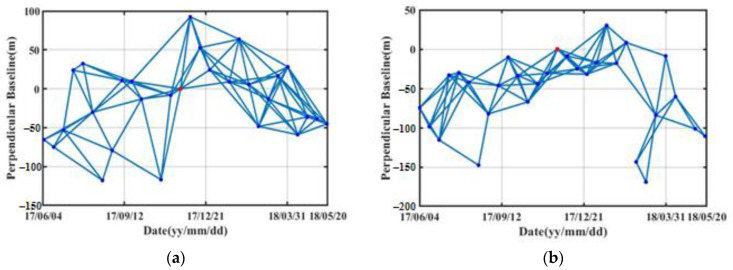
The distribution of spatial and temporal baselines in ascending (**a**) and descending (**b**) orbits. The blue dots and lines represent image acquisitions and interferometric pairs, respectively. The red dots represent the single master image used for co-registration.

In SBAS processing, a common prime image is first selected for each stack, which are 20 November 2017 for the ascending stack and 19 November 2017 for the descending stack. By setting thresholds on spatial and temporal baselines, which are 1.5% of the critical baseline and 50 days, 86 differential interferograms out of 29 SAR images are generated for the descending stack, whereas 92 differential interferograms out of 28 SAR images are generated for the ascending stack. The spatiotemporal distributions of baselines are given in Figure 7. The mosaicked DEM is used to remove topographic phase components for the interferograms. Then, the differential interferograms are unwrapped based on the Delaunay minimum cost flow method, followed by removal of orbital errors and atmospheric phase components based on iterative filtering in spatial-temporal domains. Afterwards, the time series deformation in LOS direction could be extracted using singular value decomposition (SVD).

**Figure 8:**
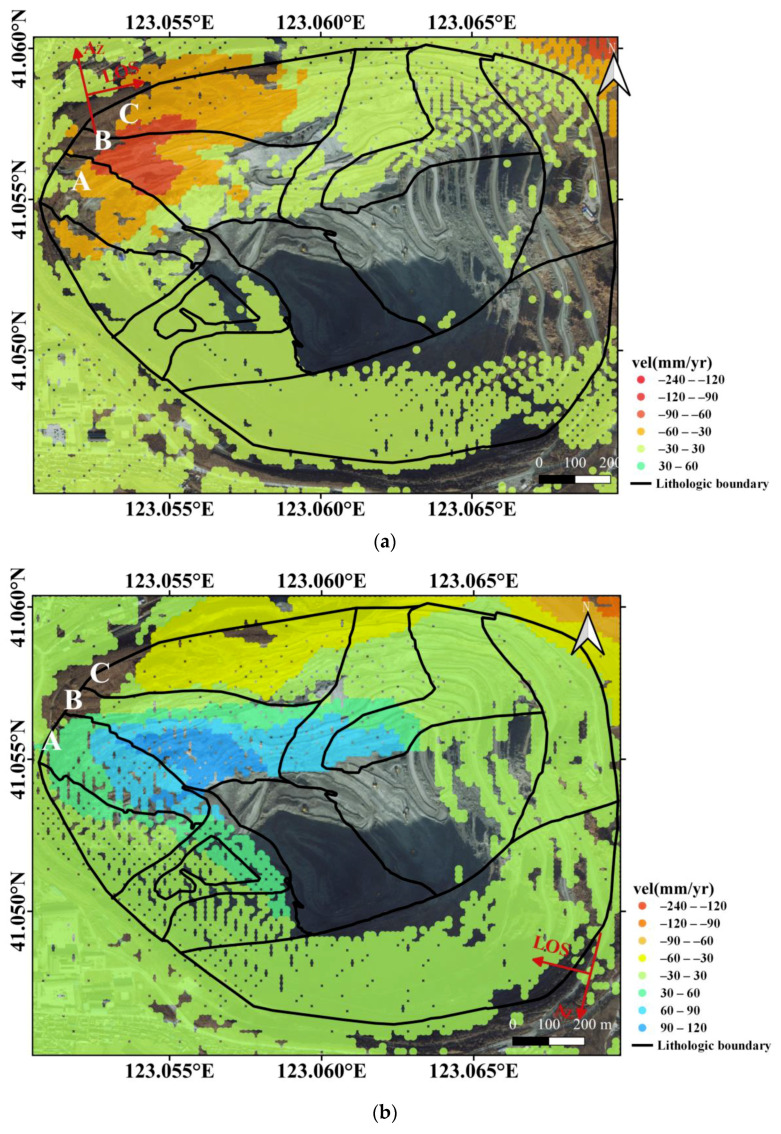
The LOS displacement velocities estimated from ascending (**a**) and descending (**b**) orbits, with google earth image as background. The red arrows refer to azimuth and LOS directions of the SAR sensor. Moreover, the black lines indicate lithological boundaries in the study area.

The LOS displacement estimated by SBAS is presented in Figure 8, where negative values indicate ground objects are moving away from the sensor, and the positive values indicate that ground objects are moving towards the sensor. The azimuth and LOS directions are presented with red arrows, respectively. As shown by Figure 8, displacements of the high and steep slopes are mainly on the northwest slope, especially in the three regions marked as A, B, and C. However, the LOS displacement velocities in descending and ascending orbits are generally opposite, with movements away from the sensor in ascending orbit and movements towards the sensor in descending orbit. This difference is generally caused by the different LOS directions of ascending and descending data.

**Figure 9:**
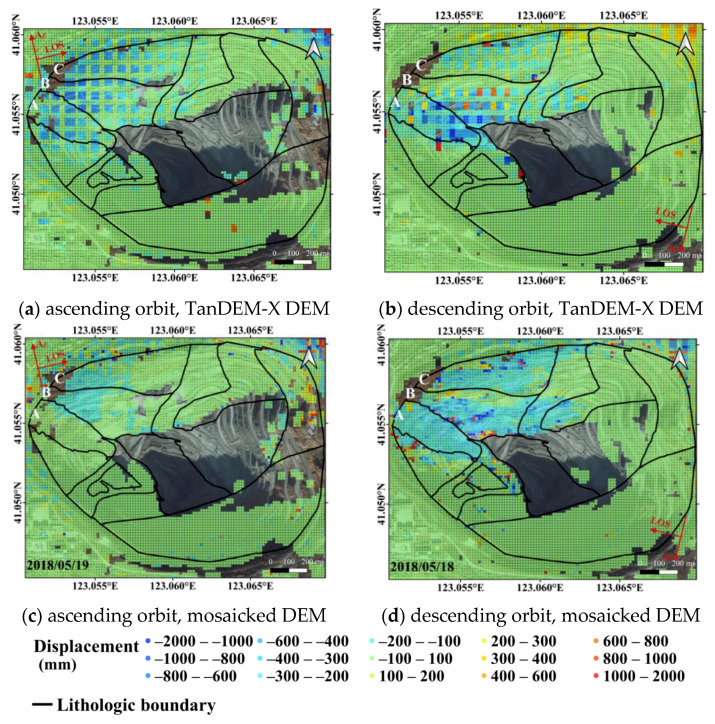
Slope displacement converted from TanDEM-X DEM and the mosaicked DEM: (**a**) ascending orbit, TanDEM-X DEM; (**b**) descending orbit, TanDEM-X DEM; (**c**) ascending orbit, mosaicked DEM; (**d**) descending orbit, mosaicked DEM.

In Section 3.2, comparison between the high resolution LiDAR DEM and TanDEM-X DEM has been given. Besides simply comparing the DEMs, conversion from LOS displacements to slope displacements are also carried out using the low resolution TanDEM-X DEM (90 m). Before conversion, the TanDEM-X DEM is first processed by the nearest neighbor interpolation in order to generate a DEM with identical spatial resolution (16.8 m) with the mosaicked DEM. Slope displacements, converted from both TanDEM-X DEM and mosaicked DEM, are shown in Figure 9, where the results from the ascending orbit are shown in the left column, and those from the descending orbit are shown in the right column. As obviously shown in Figure 9a,b, slope displacements retrieved from TanDEM-X DEM suffer from patch-like bias, which is limited by the relatively low spatial resolution and low quality of TanDEM-X DEM. On the other hand, results from the mosaicked DEM are free of patch-like bias (Figure 9c,d), and areas suffering from severe displacements are clearly described. The results from both descending and ascending orbits suggest that large displacements mainly occur in area B, although smaller displacements are detected by the ascending stack. 

### 4.2. Comparison with Ground Measurements

**Figure 10:**
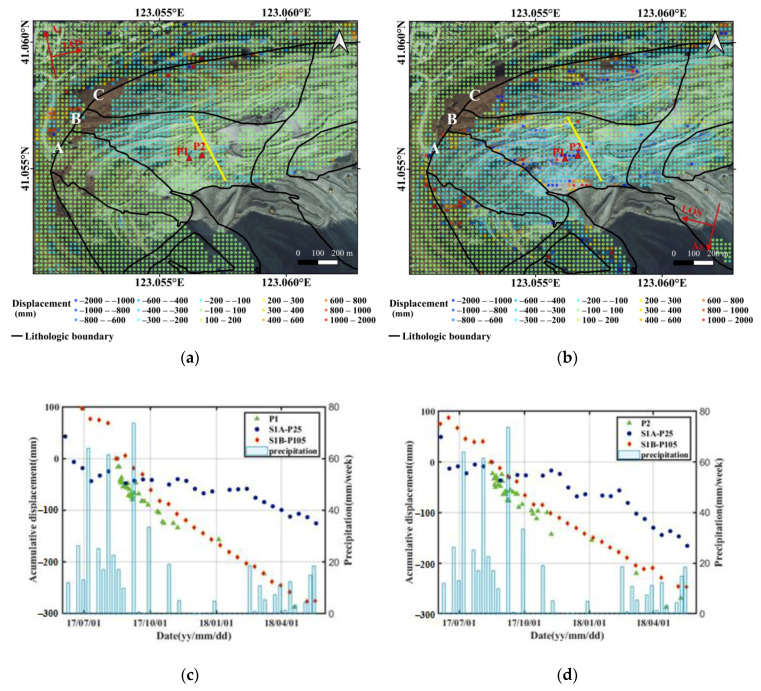
Cumulative displacement along slope direction in ascending (**a**) and descending (**b**) orbits, and slope displacement time series of ground measurement points P1 (**c**) and P2 (**d**).

During the monitoring period, continuous ground measurement on two points in area B was conducted with Leica TM30 total station from 13 August 2017 to 1 November 2018. The Leica TM30 offers 0.5″ angular accuracy and a high precision of 0.6 mm + 1 ppm to prisms. Locations of the two ground measurement points are marked as P1 and P2 in Figure 10a,b; however, they are not located on the landslide body. The slope displacement time series on the two points are depicted in Figure 10c,d, as well as the weekly precipitation. For the convenience of comparison, accumulative displacements from SAR data are registered by setting the first day of ground measurements (13 August 2017) as start time. In other words, the cumulative displacements in 13 August 2017 are set as zero in our registered time series. As shown by Figure 10c,d, results from descending orbits have shown good consistency with ground measurements, whereas a bit smaller for the ascending orbit. By comparing the slope displacement time series with weekly precipitation data, we can see that with intensive heavy rains in summer, the slope displacements show an accelerating trend, yet stabilize during the frozen period.

The root mean square errors (RMSE) of the SBAS results with reference to ground measurements on P1 and P2 are shown in Table 2. For the ascending orbit, the RMSEs of P1 and P2 are 39.6 mm and 37.2 mm, representing 12.6% and 13.1% of their accumulative displacements respectively. On the other hand, the RMSEs of P1 and P2 in descending orbit are 28.9 mm and 15.3 mm, representing 9.2% and 5.4% of their accumulative displacements respectively. The accuracies of ascending orbit data are worse than that of the descending data. This is probably because the slope aspects on P1 and P2 are almost perpendicular to LOS in ascending geometry. The slope aspects on the northwest slope are generally close to 153°, considering the heading angle of –13.539° for ascending orbit, the angle between ascending LOS and slope aspect is approximately 76°, resulting in insensitivity, and limited accuracy of the ascending orbit. However, this does not necessarily mean that ascending results over the whole mining pit are worse than the descending results. Decreased accuracy is only limited to directions almost parallel to azimuth, with RMSEs of approximately 12% to 13% on the two ground measurement points. Concerning their relatively large cumulative displacement values, the accuracies are still acceptable.

### 4.3. Microseism Events on the Northwest Slope

Many studies suggest that microseism events are indicators of rock damage, which can be accurately recorded by microseism (MS) sensors in real-time [38,39,40]. On the northwest slope, nine MS sensors were installed to continuously monitor safety of the northwest slope. From 1 September 2017 to 31 December 2017, 209 MS events were recorded. Locations, energy, and moment magnitudes of the MS events are depicted in our previous study, with reference to a typical profile, which passes through the landslide area, with elevations varying from −30 m to −294 m (as highlighted in yellow solid line in Figure 10) [31]. The slope aspect and slope angle of this profile are 153° and 47°, respectively. Since the blasting activities in Dagushan open-pit mine are well controlled, the damage caused by blasting was ignored in this study. According to the MS records, most of the events are located inside the slope and close to the surface, especially from the −66 m bench to −210 m bench. Generally, detection of MS events is an indicator of micro-fractures inside the slope, which can be considered a precursor of slope displacements. Therefore, compared to other areas, the higher density of MS events in the landslide zone means larger displacements, which is consistent with the distribution of slope displacements given by Figure 9.

In our previous study, a MS data driven damage model based on energy dissipation theory was proposed to characterize the temporal decay of rock mass mechanical parameters along mining slopes [31]. In the proposed model, rock units are automatically searched within the damage scope of MS events, and their corresponding mechanical parameters are thereafter weakened. Using the MS event data, numerical simulation based on this proposed model is conducted. The simulated damage field along the profile is presented in [31], which shows that there is almost no damage in deep part of the slope, and the damage field is mainly concentrated on shallow parts of the slope, especially in areas with elevation varies from −138 m to −210 m. Compared with undamaged areas, displacement parameters in the damaged area are generally larger, which shows very good consistency with the time series InSAR results.

## 5. Discussion on Impact Factors of the Slope Stability

### 5.1. Influence of the Geological Structure

With relatively fractured rock mass and complex engineering geological conditions, stability of the northwest slope directly affects the safety of mining production. The northwest slope in Dagushan mining stope can be roughly divided into three regions, as highlighted by Areas A, B, and C in Figure 10. According to the geological settings, area A is a low-grade wedge-shaped ore body, which is bounded by F15 and F14. Stability of the fracture zone along F14 is very poor, which threatens the railway transportation on the northwest slope. Area B is the migmatite granite group, which is composed of feldspar, quartz, muscovite, and sericite, with medium coarse-grained structure, massive, and slightly gneissic structure, or banded structure. The stability of area B is controlled by several large faults, and it is considered as a typical engineering geological model of block fractures [32]. The rock mass in area C is mainly phyllite, which is a typical cataclastic and loose engineering geological model. Concerning the very developed joints, fissures, and poor rock strength in area C, there is a high possibility of landslides under the action of precipitation [32]. However, since area C is located at the edge of the mine, with gradually reduced slope angles, instability of this area has limited threat to mining safety [32]. Therefore, area B is generally considered a potential landslide area threatening the safety of the Dagushan iron mine; displacement monitoring should be carried out continuously in this area. The monitoring results presented in this paper have also revealed instability of area B.

The east and south slopes of the Dagushan mining pit are mainly composed of granite with good lithology and structural conditions, which are generally stable. The southwest slope is a migmatite area, which is also a typical engineering geological model of block fracture. However, with less developed joints, fractures, and faults, stability of the southwest slope is therefore better than that of the northwest slope.

### 5.2. Influence of Precipitation

Previous studies have shown that most slope instabilities are caused by precipitation, in addition to geological settings and lithology. Landslides in open-pit mines usually occur during rainy season or thawing period, during which the rich water can change rock mechanical properties and reduce rock mechanical strength. Infiltration and accumulation of meteoric water, as well as activity of groundwater, can seriously reduce the stability of slopes, which are composed of loose sediments and weathered rocks. The water that seeps into structural planes, especially into weak structural planes, will significantly reduce the shear strength, increase the sliding force, and eventually form structural failures of the rock mass [41,42]. As shown in Figure 10c,d, after a large amount of precipitation (from June to August every year), the displacements of both points accelerated.

Up to 69% of the rock mass in the fracture zone of the northwest slope is composed of clay minerals. Mechanical connection between different rock blocks will be weakened by the water rock reaction of clay minerals. As a result, the integrity and stability of rock mass will decline, as well as the stability of single rock blocks. Under the influence of rainstorm, the strength of fracture zones decreases as water absorption rate increases. In 27 May 2018, due to the double influence of lithology and heavy rain, a surface landslide occurred in the fracture zone of northwest slope, resulting in a large number of gravel rolling and road blocked, which affected the transportation of mineral ores [43].

### 5.3. Influence of Slope Aspects and Imaging Orbit

As shown in Table 2, accuracies of P1 and P2 in ascending geometry are both worse than those in descending orbit. This is because aspects of the northwest slope are nearly parallel to the azimuth and perpendicular to the LOS in ascending orbit. This particular geometry could lead to insensitivity and reduced accuracy for slope displacement estimation. Similar problems also exist in descending geometry, which means in slope aspects nearly parallel to the descending azimuth also suffer from reduced sensitivity and accuracy. However, with assistance of the precise DEM, the estimated slope displacements are still acceptable. For example, the RMSEs for P1 and P2 in ascending orbit are approximately 12.6–13.1% of the accumulative displacement. Besides simply converting LOS displacements of a single imaging orbit to slope direction, fusion of results from both ascending and descending orbits is also a way of improving displacement estimation accuracy, which would be exploited in the future.

## 6. Conclusions

In this manuscript, a precise digital elevation model (DEM) based method that converts LOS displacement to slope direction is proposed, and a case study over the high and steep slopes of the Dagushan open pit iron mine is carried out. The precise DEM is generated by mosaicking TanDEM with LiDAR DEM. Then, the mosaicked DEM is innovatively used to assist LOS displacement estimation with small baseline subset analysis on two stacks of sentinel-1 images. Then, the slope displacements over the Dagushan open pit mine are converted from LOS measurements with the proposed method. By comparing the results with ground measurement points, relative RMSE of approximately 12.6–13.1% for the ascending orbit and 5.4–9.2% for the descending orbit are achieved in our study area. The distribution of slope displacements has also shown good agreement with the microseism results. Both results show that the largest slope displacements occur on the slope part, with elevations varying from −138 m to −210 m, which corresponds to the landslide area. Moreover, there is a certain correlation with precipitation. The outcome of this article shows that rock mass structure, lithology, and precipitation are main factors affecting the stability of high and steep slopes in open-pit mines.

## Figures and Tables

**Table 1:** Detailed parameters of the synthetic aperture radar (SAR) datasets.

Sensor	Orbit Path	Number of Images	Temporal Coverage	Orbit Direction	Heading (°)	Incidence Angle (°)
Sentinel-1A	25	28	5 June 2017–19 May 2018	Ascending	−13.539	33.726
Sentinel-1B	105	29	4 June 2017–18 May 2018	Descending	−166.421	43.890

**Table 2:** Root mean square error (RMSE) of slope displacements on P1 and P2, with reference to ground measurements (mm).

Point ID	Accumulative Displacement	RMSE Ascending	Percentage Ascending	RMSE Descending	Percentage Descending
**P1**	−314.2	39.6	12.6%	28.9	9.2%
**P2**	−284.6	37.2	13.1%	15.3	5.4%
